# Banxia Baizhu Tianma Decoction for Essential Hypertension: A Systematic Review of Randomized Controlled Trials

**DOI:** 10.1155/2012/271462

**Published:** 2012-12-20

**Authors:** Xingjiang Xiong, Xiaochen Yang, Wei Liu, Bo Feng, Jizheng Ma, Xinliang Du, Pengqian Wang, Fuyong Chu, Jun Li, Jie Wang

**Affiliations:** ^1^Department of Cardiology, Guang′anmen Hospital, China Academy of Chinese Medical Sciences, Beixiange 5, Xicheng District, Beijing 100053, China; ^2^Department of Gastroenterology, Guang′anmen Hospital, China Academy of Chinese Medical Sciences, Beijing 100053, China; ^3^Graduate School, China Academy of Chinese Medical Sciences, Beijing 100700, China; ^4^Department of Endocrinology, Traditional Chinese Medicine Hospital of Mentougou District, Beijing 102300, China; ^5^Department of Cardiology, Traditional Chinese Medicine Hospital of Beijing, Beijing 100010, China

## Abstract

*Objectives*. To assess the current clinical evidence of Banxia Baizhu Tianma Decoction (BBTD) for essential hypertension (EH). *Search Strategy*. Electronic databases were searched until July 2012. *Inclusion Criteria*. We included randomized clinical trials testing BBTD against placebo, antihypertensive drugs, or combined with antihypertensive drugs against antihypertensive drugs. *Data Extraction and Analyses*. Study selection, data extraction, quality assessment, and data analyses were conducted according to Cochrane standards. *Results*. 16 randomized trials were included. Methodological quality of the included trials was evaluated as generally low. 2 trials compared prescriptions based on BBTD using alone with antihypertensive drugs. Meta-analysis showed no significant effect of modified BBTD compared with captopril in systolic blood pressure (MD: −0.75 (−5.77, 4.27); *P* = 0.77) and diastolic blood pressure (MD: −0.75 (−2.89, 1.39); *P* = 0.49). 14 trials compared the combination of BBTD or modified BBTD plus antihypertensive drugs with antihypertensive drugs. Meta-analysis showed there are significant beneficial effect on systolic blood pressure in the combination group compare to the antihypertensive drugs (MD: −4.33 (−8.44, −0.22); *P* = 0.04). The safety of BBTD is uncertain. *Conclusions*. There is encouraging evidence of BBTD for lowering SBP, but evidence remains weak. Rigorously designed trials are warranted to confirm these results.

## 1. Introduction

Hypertension is an increasingly important medical and public health issue, which could lead to severe complications [1]. High blood pressure is a major, independent risk factor for cardiovascular disease (CVD). The relationship between blood pressure (BP) and risk of CVD events is continuous, consistent, and independent of other risk factors. The higher the BP, the greater is the chance of heart attack, heart failure, stroke, and kidney diseases. The prevention and management of hypertension are major public health challenges. Much of hypertension, cardiovascular, and cerebrovascular diseases would be preventable if the rise in BP with age could be prevented or diminished [2]. 

Complementary and alternative medicine (CAM) is becoming increasingly popular and frequently used among patients with CVD [3–7]. Approximately 50% of US residents use some form of alternative medicine; 10% use it for their children [8]. Recent researches showed that CAM (also integrative medicine) could contribute to blood pressure control [9–12]. Chinese medicine (CM) [13, 14], including herbal medicine, acupuncture, moxibustion, and cupping *Tai chi* and *Qigong*, as one of the most important parts in CAM, is thought to be effective for the treatment of essential hypertension [15–18]. It has been considered as an effective adjunct treatment by either physicians or patients in China. More and more patients firstly select the combination therapy, just CM combined with antihypertensive drugs, for better efficacy both in BP and clinical symptom such as headache, neck and shoulder pain, dizziness, and fatigue. For seeking the best evidence of CM in making decisions for hypertensive patients, an increasing number of systematic reviews (SR) and meta-analysis have been conducted to assess the efficiency of CM for hypertension [19–24]. It is found out that CM could contribute to lower BP smoothly, recover the circadian rhythm of BP, and improve symptoms and signs especially [25]. And the efficacy of CM for treating hypertension is suggested by a large number of published case series and randomized trials [26, 27], although some trials have demonstrated negative results [28, 29]. Mechanistic studies have demonstrated that the antihypertensive effect is related to activation of endothelial nitric oxide synthase (eNOS) and inducible nitric oxide synthase (iNOS) [30, 31], regulation of vascular endothelium function [26, 32], inhibiting proliferation of adventitial fibroblasts and collagen synthesis [33], inhibition of vascular smooth muscle cell proliferation [34], and so forth. A series of Chinese herbs have been authorized recommended by the Chinese government in Pharmacopoeia of the People's Republic of China (2010 edition).

Banxia baizhu tianma decoction (BBTD), containing eight commonly used herbs (*Pinellia ternata*, atractylodes macrocephala, *Gastrodia elata*, tangerine peel, poria cocos, *Glycyrrhiza*, ginger, and red jujube), is a classical Chinese herbal formula noted in *Medical Revelations *in *Qing* dynasty. It has been widely used to treat hypertension-related symptoms in clinical practice for centuries in China [25]. The most common symptoms include headache, dizziness, nausea, and vomiting, which belong to the liver yang hyperactivity and fluid retention syndrome [25]. The mechanism of the prescription maybe calming liver, suppressing liver yang hyperactivity, dissipating excessive fluid, and expelling phlegm according to the theory of TCM. Recently, modern researches showed that BBTD have potential effect of lowing BP *in vitro* and *in vivo* [25, 35–38]. Biochemically, BBTD also showed good effect in improving the mesenteric endothelial dysfunction and the hemodynamic parameters, inhibiting the expression of nitric oxide (NO) and interleukin-1 (IL-1), decreasing serum levels of total cholesterol (TC), triglycerides (TGs), and low-density lipoprotein-cholesterol (LDL-C), regulating rennin-angiotensin system (RAS), and improving the oxidative stress state, so as to lower the arterial pressure [35–38]. 

Currently, BBTD used alone or combined with antihypertensive drugs has been widely used as an alternative and effective method for the treatment of essential hypertension in China. And until now a number of clinical studies of BBTD reported the clinical effect ranging from case reports and case series to controlled observational studies and randomized clinical trials. However, there is no critically appraised evidence such as systematic reviews or meta analyses on potential benefits and harms of BBTD for essential hypertension to justify their clinical use and their recommendation. This paper aims to assess the current clinical evidence of BBTD for essential hypertension.

## 2. Methods

### 2.1. Database and Search Strategies

Literature searches were conducted in Chinese National Knowledge Infrastructure (CNKI), Chinese Scientific Journal Database (VIP), Chinese Biomedical Literature Database (CBM), PubMed, EMBASE, and the Cochrane Central Register of Controlled Trials (CENTRAL) in the Cochrane Library (July 2012). We also searched the reference list of retrieved papers. Databases in Chinese were searched to retrieve the maximum possible number of trials of BBTD for essential hypertension because BBTD is mainly used and researched in China. All of those searches ended on 3 July, 2012. Ongoing registered clinical trials were searched in the website of Chinese clinical trial registry (http://www.chictr.org/) and international clinical trial registry by US National Institutes of Health (http://clinicaltrials.gov/). The following search terms were used individually or combined: “hypertension,” “essential hypertension,” “banxia baizhu tianma decoction,” “clinical trial,” and “randomized controlled trial.” The bibliographies of included studies were searched for additional references. 

### 2.2. Inclusion Criteria

 All the parallel randomized controlled trials (RCTs) of all the prescriptions based on “banxia baizhu tianma decoction” compared with antihypertensive drugs in patients with hypertension were included. RCTs combined banxia baizhu tianma decoction with antihypertensive drugs compared with antihypertensive drugs and all the modified banxia baizhu tianma decoction were included as well. According to the principle of the similarity of traditional Chinese medicine (TCM) formula [39], the number of modified herbs should not be more than 4, so that to ensure the similarity is greater than or equal to 0.5. And the key herbs in the modified banxia baizhu tianma decoction should include *Pinellia ternata*, atractylodes macrocephala, *Gastrodia elata*, and poria cocos, according to the theory of TCM. There were no restrictions on population characteristics, language and publication type. The main outcome measure was blood pressure. Duplicated publications reporting the same groups of participants were excluded.

### 2.3. Data Extraction and Quality Assessment

Two authors conducted the literature searching (Xiong and Yang), study selection (Xiong and Wang), and data extraction (Xiong and Li) independently. The extracted data included authors, title of study, year of publication, study size, age and sex of the participants, details of methodological information, name and component of Chinese herbs, treatment process, details of the control interventions, outcomes, and adverse effects for each study. Disagreement was resolved by discussion and reached consensus through a third party (J. Wang). 

The methodological quality of trials was assessed independently using criteria from the Cochrane Handbook for Systematic Review of Interventions, Version 5.1.0 (Xiong and Yang) [56]. The items included random sequence generation (selection bias), allocation concealment (selection bias), blinding of participants and personnel (performance bias), blinding of outcome assessment (detection bias), incomplete outcome data (attrition bias), selective reporting (reporting bias), and other bias. The quality of all the included trials was categorized to low/unclear/high risk of bias (“yes” for a low of bias, “no” for a high risk of bias, “unclear” otherwise). Then trials were categorized into three levels: low risk of bias (all the items were in low risk of bias), high risk of bias (at least one item was in high risk of bias), unclear risk of bias (at least one item was in unclear). 

### 2.4. Data Synthesis

RevMan 5.1 software provided by the Cochrane Collaboration was used for data analyses. Continuous outcome will be presented as mean difference (MD) and its 95% CI. Heterogeneity was recognized significant when *I* ^2^ ≥ 50%. Fixed-effects model was used if there is no significant heterogeneity of the data; random-effects model was used if significant heterogeneity existed (50% < *I* ^2^ < 85%). Publication bias would be explored by funnel plot analysis if sufficient studies were found. 

## 3. Result

### 3.1. Description of Included Trials

**Figure 1:**
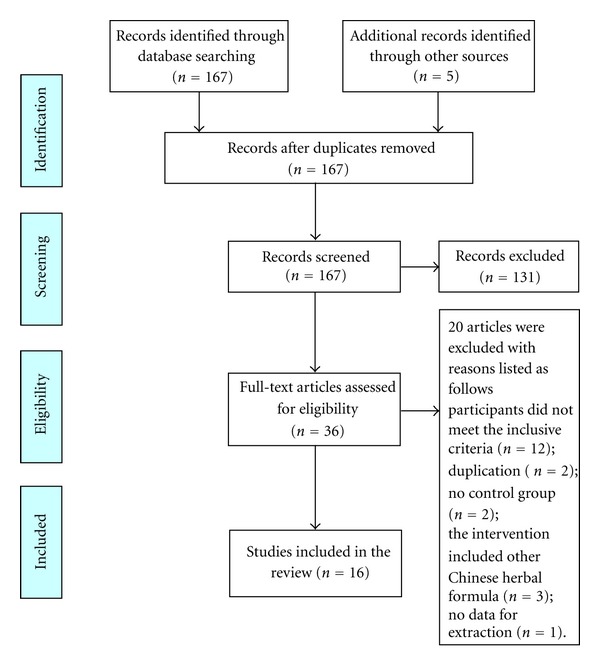
PRISMA 2009 flow diagram.

A flow chart depicted the search process and study selection (as shown in Figure 1). After primary searches from the databases, 167 articles were screened. After reading the titles and abstracts, 131 articles of them were excluded. Full texts of 36 articles were retrieved, and 20 articles were excluded with reasons listed as follows: participants did not meet the inclusive criteria (*n* = 12), duplication (*n* = 2), no control group (*n* = 2), the intervention included other Chinese herbal formula (*n* = 3), and no data for extraction (*n* = 1). In the end, 16 RCTs [40–55] were included. All the RCTs were conducted in China and published in Chinese. The characteristics of included trials were listed in Table 1. 

1424 patients with essential hypertension were included. There was a wide variation in the age of subjects (19–78 years). Sixteen (16) trials specified five diagnostic criteria of hypertension, five trials [40, 41, 45, 50, 52] used Chinese Guidelines for the Management of Hypertension-2005 (CGMH-2005), five trials [43, 44, 49, 53, 55] used 1999 WHO-ISH guidelines for the management of hypertension (1999 WHO-ISH GMH), two trials [46, 47] used China Guidelines on Prevention and Management of High Blood Pressure-2004 (CGPMHBP-2004), one trial [51] used Chinese Guidelines for the Management of Hypertension-1999 (CGMH-1999), one trial [51] used the Sixth Report of the Joint National Committee on Prevention, Detection, Evaluation, and Treatment of High Blood Pressure (JNC-VI), and two trials [42, 48] only demonstrated patients with essential hypertension. Sixteen (16) trials specified three diagnostic criteria of abundant phlegm-dampness syndrome in TCM, nine trials [41, 45, 46, 49–53, 55] used Guidelines of Clinical Research of New Drugs of Traditional Chinese Medicine (GCRNDTCM), one trial [42] used Convention of Diagnosis and Treatment of Disease and Syndrome in Shanghai (CDTDSS), one trial [47] used Chinese internal medicine (CIM), two trials [44, 48] only demonstrated patients with abundant phlegm-dampness syndrome in TCM, and three trials [40, 43, 54] did not report any TCM diagnostic criteria. 

Interventions included all the prescriptions based on “banxia baizhu tianma decoction” alone, or combined with antihypertensive drugs. The controls included antihypertensive drugs alone. Two trials investigated the prescriptions based on “banxia baizhu tianma decoction” used alone [40, 53] versus antihypertensive drugs, and the rest fourteen trials [41–52, 54, 55] compared the prescriptions based on “banxia baizhu tianma decoction” plus antihypertensive drugs versus antihypertensive drugs.

The total treatment duration ranged from 7 days to 3 months. The variable prescriptions are presented in Table 1. The different compositions of Chinese herbal formula BBTD are presented in Table 2. All of the 16 trials used the BP as the outcome measure. Adverse effect was described in detail. 

### 3.2. Methodological Quality of Included Trials

The majority of the included trials were assessed to be of general poor methodological quality according to the predefined quality assessment criteria (Table 3). The randomized allocation of participants was mentioned in all trials; however, only 5 trials stated the methods for sequence generation including random number table [40, 44, 46, 54] and drawing [48]. Insufficient information was provided to judge whether or not it was conducted properly. Allocation concealment, blinding of participants and personnel, and blinding of outcome assessment were not mentioned in all trials. None of trials reported dropout or withdraw. None of trials had a pretrial estimation of sample size. All the trials did not mention followup. We tried to contact the author for further information; however, no information has been provided to date.

### 3.3. Effect of the Interventions

#### 3.3.1. “Banxia Baizhu Tianma Decoction” versus Antihypertensive Drugs (Western Medicine)

Two trials [40, 53] compared prescriptions based on BBTD used alone with antihypertensive drugs. A change in blood pressure was reported in all the two RCTs [40, 53]. One trial [53] showed the homogeneity in the consistency of the trial results. Thus, fixed-effects model should be used for statistical analysis. The meta-analysis showed no significant effect of modified BBTD compared with captopril alone in systolic blood pressure (MD: −0.75 (−5.77, 4.27); *P* = 0.77) and diastolic blood pressure (MD: −0.75 (−2.89, 1.39); *P* = 0.49) (Tables 4 and 5). 

#### 3.3.2. “Banxia Baizhu Tianma Decoction” Plus Antihypertensive Drugs versus Antihypertensive Drugs

Fourteen trials [41–52, 54, 55] compared the combination of BBTD or modified BBTD plus antihypertensive drugs with antihypertensive drugs. A change in blood pressure was reported in all the included RCTs.

**Systolic Blood Pressure (SBP)**

The 3 independent trials [41, 46, 51] did not show homogeneity in the trial results, chi-square = 7.18, (*P* = 0.03); *I* ^2^ = 72%. Thus, random-effects model should be used for statistical analysis. The meta-analysis showed that there are significant beneficial effect on the combination group compared to the antihypertensive drugs used alone (MD: −4.33 (−8.44, −0.22); *P* = 0.04) (Table 4).

**Diastolic Blood Pressure (DBP)**

Three trials [41, 46, 51] did not show homogeneity in the trial results, chi-square = 6.87, (*P* = 0.03); *I* ^2^ = 71%. Thus, random-effects model should be used for statistical analysis. The meta-analysis showed that there are no significant beneficial effect on the combination group compare to the antihypertensive drugs used alone (MD: −1.57 (−4.54, 1.40); *P* = 0.30) (Table 5).

### 3.4. Publication Bias

The number of trials was too small to conduct any sufficient additional analysis of publication bias. 

### 3.5. Adverse Effect

Four out of sixteen trials mentioned the adverse effect [40, 44, 45, 50]. Four trials reported five specific symptoms including headache, distending feeling in head, palpitations, drowsiness, and fatigue. Among them, no adverse events were found in two trials [44, 45]. One trial reported adverse effect in enalapril group including headache, palpitations, drowsiness, and fatigue [40]. One trial mentioned adverse effect both in modified BBTD plus nifedipine sustained release tablets group and nifedipine sustained release tablets group including distending feeling in head [50]. 

## 4. Discussion

Based on the paper and meta-analyses of the outcome on either SBP or DBP, BBTD may have positive effects for lowing BP. BBTD as an adjunctive treatment to antihypertensive drugs significantly lowered SBP in patients with hypertension. However, according to potential publication bias and low-quality trials, available data are not adequate to draw a definite conclusion of BBTD for essential hypertension. And the positive findings should be interpreted conservatively. 

Several limitations should be considered before accepting the findings of this paper. First, the quality of the included RCTs is generally low. Sixteen trials included in this paper had risk of bias in terms of design, reporting, and methodology. They provided only inadequate reporting of study design, allocation sequence, allocation concealment, blinding, intention to treat analysis, and dropouts account in the majority of trials. Randomization was mentioned but without further details, which do not allow a proper judgment of the conduct of the trials. All the trials did not describe the blinding in details. It directly led to performance bias and detection bias due to patients and researchers being aware of the therapeutic interventions for the subjective outcome measures. All the sixteen RCTs prohibited us to perform meaningful sensitivity analysis. All the included trials were not multicenter, large-scale RCTs. If poorly designed, all the trials would show larger differences compared with well designed trials. 

Second, all the sixteen trials did not report the adverse effect of banxia baizhu tianma decoction. Therefore, a conclusion about the safety of BBTD cannot be made clearly. In China, it is widely believed that it is safe to use herbal medicines for various diseases. With more and more reports of adverse effects of Chinese herbal medicines, the safety of Chinese herbs and formulae needs to be monitored rigorously and reported appropriately in the future clinical trials.

Third, Vickers et al. demonstrated that some countries, for example, China, generate virtually no “negative” studies at all [57]. In other words, publication and other biases may play an important role. We only identified and included trials published in Chinese after conducting comprehensive searches. Most of the trials are small sample with positive findings. We tried to avoid language bias and location bias, but we cannot exclude potential publication bias.

Fourth, it is pointed out that, lacking Chinese medicine (CM) pattern criteria (also called syndrome or zheng) become the key issue both for RCT and clinical practice [58–60]. For example, receiving CM or conventional therapies in patients with the same disease respectively, conventional treatment tends to produce a better curative effect than CM [61–64]. This should be the major reason why the RCTs failed to evaluate the real efficacy of CM. In this systematic review, three out of the sixteen trials [40, 43, 54] did not report the TCM diagnostic criteria. Two trials [44, 48] reported the TCM diagnostic criteria but without further details. Therefore, further clinical trials should be conducted with clear TCM diagnostic criteria.

In conclusion, there is some encouraging evidence of BBTD for lowering SBP, but the evidence remains weak due to poor methodological quality of including studies. Rigorously designed trials seem to be warranted to confirm the results.

## Figures and Tables

**Table 1:** Characteristics and methodological quality of included studies.

Study ID	Sample	Diagnosis standard	Intervention	Control	Course (week)	Outcome measure
Zheng, 2011 [40]	60	CGMH-2005	Modified BBTD (600 mL/d^#^)	Enalapril (10 mg qd)	3	BP; adverse effect
Xiong, 2010 [41]	60	CGMH-2005; GCRNDTCM	Modified BBTD (250 mL/d^#^) plus L-amlodipine (2.5 mg qd)	L-amlodipine (2.5 mg qd)	4	BP
Chen et al., 2005 [42]	70	Hypertension diagnostic criteria (unclear); CDTDSS	Modified BBTD (240 mL/d^#^) plus nitrendipine (no detailed information)	Nitrendipine (no detailed information)	8	BP
Wang, 2001 [43]	100	1999 WHO-ISH GMH	Modified BBTD (1 dose/d^#^) plus captopril (25–37.5 mg tid)	Captopril (25–37.5 mg tid)	4	BP
Che et al., 2011 [44]	60	1999 WHO-ISH GMH; TCM diagnostic criteria (unclear)	Modified BBTD (400 mL/d^#^) plus nifedipine controlled release tablet (10 mg bid)	Nifedipine controlled release tablet (10 mg bid)	4	BP; adverse effect
Jin, 2011 [45]	60	CGMH-2005; GCRNDTCM	BBTD plus antihypertensive drugs (400 mL/d^#^)	Antihypertensive drugs (no detailed information)	6	BP; adverse effect
Chen, 2007 [46]	120	CGPMHBP-2004; GCRNDTCM	Modified BBTD (1 dose/d^#^) plus losartan (50 mg qd)	Losartan (50 mg qd)	12	BP
Guo, 2009 [47]	94	CGPMHBP-2004; CIM	Modified BBTD (400–500 mL/d^#^) plus compound reserpine-triamterene tablets (1 pill, qd)	Compound reserpine-triamterene tablets (1 pill, qd)	4	BP
Li, 2011 [48]	139	Hypertension and TCM diagnostic criteria (unclear)	Modified BBTD (450 mL/d^#^) plus felodipine sustained release tablets (1 pill, bid)/levamlodipine besylate tablets (1 pill, qd)	Felodipine sustained release tablets (1 pill, bid)/levamlodipine besylate tablets (1 pill, qd)	12	BP
Guo et al., 2006 [49]	116	1999 WHO—ISH GMH; GCRNDTCM	Modified BBTD (250 mL/d^#^) plus levamlodipine besylate tablets (5 mg qd)	Levamlodipine besylate tablets (5 mg qd)	2	BP
Zhou, 2008 [50]	102	CGMH-2005; GCRNDTCM	Modified BBTD (400 mL/d^#^) plus nifedipine sustained release tablets (10 mg bid)	Nifedipine sustained release tablets (10 mg bid)	4	BP; adverse effect
Wu et al., 2007 [51]	87	CGMH-1999; GCRNDTCM	Modified BBTD (1 dose/d^#^) plus antihypertensive drugs (no detailed information)	Antihypertensive drugs (no detailed information)	8	BP
Lei and Lin, 2009 [52]	114	CGMH-2005; GCRNDTCM	BBTD (400 mL/d^#^) plus benazepril (10 mg qd)	Benazeprill (10 mg qd)	4	BP
Liu et al., 2007 [53]	80	1999 WHO—ISH GMH; GCRNDTCM	Modified BBTD (300 mL/d^#^)	Captopril (12.5 mg bid)	4	BP
Zhang, 2002 [54]	80	JNC-VI	Modified BBTD (1 dose/d^#^) plus felodipine (5 mg qd)/hydrochlorothiazide (12.5 mg bid)	Felodipine (5 mg qd)/hydrochlorothiazide (12.5 mg bid)	1	BP
Wang, 2005 [55]	82	1999 WHO—ISH GMH; GCRNDTCM	Modified BBTD (1 dose/d^#^) plus captopril (no detailed information)	Captopril (no detailed information)	4	BP

**Table 2:** Composition of formula.

Study ID	Formula	Composition of formula
Zheng, 2011 [40]	Modified BBTD	*Pinellia ternate* 9 g, atractylodes macrocephala 15 g, *Gastrodia elata* 10 g, tangerine peel 10 g, poria cocos 10 g, *Glycyrrhiza* 4 g, ginger 2 pieces, red jujube 5, grass leaved sweetflag 10 g, ligusticum chuanxiong hort 9 g, alisma orientalis 15 g, and *Grifola umbellata* 10 g
Xiong, 2010 [41]	Modified BBTD	*Pinellia ternate* 12 g, atractylodes macrocephala 15 g, *Gastrodia elata* 15 g, tangerine peel 12 g, poria cocos 12 g, alisma orientalis 15 g, plantain seed 15 g, bamboo bark 9 g, villous amomum fruit 3 g, *Pinellia pedatisecta* Schott 12 g, grass leaved sweetflag 15 g, ginger 9 g, red jujube 5, and *Glycyrrhiza* 6 g
Chen et al., 2005 [42]	Modified BBTD	*Pinellia ternate* 6 g, *Gastrodia elata* 9 g, atractylodes macrocephala 9 g, poria cocos 12 g, tangerine peel 12 g, *Pinellia pedatisecta* Schott 12 g, fructus aurantii 12 g, *Glycyrrhiza* 6 g
Wang, 2001 [43]	Modified BBTD	*Pinellia ternate* 15 g, atractylodes macrocephala 12 g, *Gastrodia elata* 15 g, tangerine peel 12 g, poria cocos 12 g, alisma orientalis 15 g, *Uncaria* 15 g (put in later), abalone shell 15 g (decocting first), ginger 15 g, jujube 5, and *Glycyrrhiza* 6 g
Che et al., 2011 [44]	Modified BBTD	*Pinellia ternate* 15 g, atractylodes macrocephala 25 g, *Gastrodia elata* 10 g, tangerine peel 10 g, poria cocos 10 g, kudzu root 10 g, *Sophora* flower 15 g, cassia seed 10 g, hawthorn 15 g, and *Glycyrrhiza* 5 g
Jin, 2011 [45]	BBTD	*Pinellia ternate* 10 g, atractylodes macrocephala 10 g, *Gastrodia elata* 10 g, tangerine peel 10 g, poria cocos 15 g, *Glycyrrhiza* 5 g, ginger 10 g, and jujube 10 g
Chen, 2007 [46]	Modified BBTD	*Pinellia ternate* 9 g, atractylodes macrocephala 12 g, *Gastrodia elata* 6 g, tangerine peel 10 g, poria cocos 15 g, alisma orientalis 10 g, hawthorn 10 g, cassia seed 15 g, grass leaved sweetflag 6 g, ligusticum chuanxiong hort 6 g, *Salvia miltiorrhiza* 12 g, and *Glycyrrhiza* 5 g
Guo, 2009 [47]	Modified BBTD	*Pinellia ternate* 12 g, atractylodes macrocephala 15 g, *Gastrodia elata* 10 g, tangerine peel 9 g, poria cocos 10 g, ligusticum chuanxiong hort 10 g, officinal magnolia bark 6–10 g, chrysoidine 9 g, grass leaved sweetflag 10 g, curcuma longa 10 g, ginger 3 pieces, and jujube 3
Li, 2011 [48]	Modified BBTD	*Pinellia ternate* 10 g, atractylodes macrocephala 10 g, *Gastrodia elata* 10 g, tangerine peel 12 g, poria cocos 15 g, citrus aurantium 10 g, bamboo bark 10 g, and *Glycyrrhiza* 6 g
Guo et al., 2006 [49]	Modified BBTD	*Pinellia ternate* 18 g, atractylodes macrocephala 12 g, *Gastrodia elata* 18 g, tangerine peel 12 g, poria cocos 15 g, grass leaved sweetflag 15 g, *Eucommia ulmoides Oliv.* 15 g, *Prunella vulgaris* 12 g, *Glycyrrhiza* 6 g, and jujube 5
Zhou, 2008 [50]	Modified BBTD	*Pinellia ternate* 12 g, atractylodes macrocephala 12 g, *Gastrodia elata* 6 g, tangerine peel 9 g, poria cocos 12 g, bamboo bark 9 g, *Glycyrrhiza* 6 g, villous amomum fruit 3 g, ginger 3 g, and jujube 5
Wu et al., 2007 [51]	Modified BBTD	*Pinellia ternate* 10 g, atractylodes macrocephala 10 g, *Gastrodia elata* 10 g, tangerine peel 10 g, poria cocos 15 g, bamboo bark 10 g, *Coix lacryma-jobi* 20 g, *Glycyrrhiza* 3 g, and ginger 3 pieces
Lei and Lin, 2009 [52]	BBTD	*Pinellia ternate* 12 g, atractylodes macrocephala 12 g, *Gastrodia elata* 15 g, tangerine peel 9 g, poria cocos 12 g, *Glycyrrhiza* 6 g, ginger 3 g, and jujube 5
Liu et al., 2007 [53]	Modified BBTD	*Pinellia ternate* 9 g, atractylodes macrocephala 15 g, *Gastrodia elata* 6 g, tangerine peel 6 g, poria cocos 6 g, *Glycyrrhiza* 5 g, angelica sinensis 10 g, white peony root 10 g, lotus leaf 15 g, and alisma orientalis 15 g
Zhang, 2002 [54]	Modified BBTD	*Pinellia ternate* 15 g, atractylodes macrocephala 15 g, *Gastrodia elata* 12 g, tangerine peel 12 g, poria cocos 12 g, *Glycyrrhiza* 10 g, plantain seed 15 g, and *Loranthus parasiticus* 15 g
Wang, 2005 [55]	Modified BBTD	*Pinellia ternate* 10 g, atractylodes macrocephala 15 g, *Gastrodia elata* 15 g, tangerine peel 15 g, poria cocos 30 g, hawthorn 15 g, and grass leaved sweetflag 15 g

**Table 3:** Quality assessment of included randomized controlled trials.

Included trials	Random sequence generation	Allocation concealment	Blinding of participants and personnel	Blinding of outcome assessment	Incomplete outcome data	Selective reporting	Other sources of bias	Risk of bias
Zheng, 2011 [40]	Table of random number	Unclear	Unclear	Unclear	No	No	Unclear	Unclear
Xiong, 2010 [41]	Unclear	Unclear	Unclear	Unclear	Yes	No	Unclear	High
Chen et al., 2005 [42]	Unclear	Unclear	Unclear	Unclear	Yes	No	Unclear	High
Wang, 2001 [43]	Unclear	Unclear	Unclear	Unclear	Yes	No	Unclear	High
Che et al., 2011 [44]	Table of random number	Unclear	Unclear	Unclear	No	No	Unclear	Unclear
Jin, 2011 [45]	Unclear	Unclear	Unclear	Unclear	No	No	Unclear	High
Chen, 2007 [46]	Table of random number	Unclear	Unclear	Unclear	Yes	No	Unclear	Unclear
Guo, 2009 [47]	Unclear	Unclear	Unclear	Unclear	Yes	No	Unclear	High
Li, 2011 [48]	Drawing	Unclear	Unclear	Unclear	Yes	No	Unclear	Unclear
Guo et al., 2006 [49]	Unclear	Unclear	Unclear	Unclear	Yes	No	Unclear	High
Zhou, 2008 [50]	Unclear	Unclear	Unclear	Unclear	No	No	Unclear	High
Wu et al., 2007 [51]	Unclear	Unclear	Unclear	Unclear	Yes	No	Unclear	High
Lei and Lin, 2009 [52]	Unclear	Unclear	Unclear	Unclear	Yes	No	Unclear	High
Liu et al., 2007 [53]	Unclear	Unclear	Unclear	Unclear	Yes	No	Unclear	High
Zhang, 2002 [54]	Table of random number	Unclear	Unclear	Unclear	Yes	No	Unclear	High
Wang, 2005 [55]	Unclear	Unclear	Unclear	Unclear	Yes	No	Unclear	High

**Table 4:** Analyses of systolic blood pressure.

Trials		MD (95% CI)	*P* value
BBTD versus antihypertensive drugs			
Modified BBTD versus captopril	1	−0.75 (−5.77, 4.27)	0.77

*Meta-analysis *	1	−0.75 (−5.77, 4.27)	0.77

BBTD plus antihypertensive drugs versus antihypertensive drugs			
Modified BBTD plus L-amlodipine versus L-amlodipine	1	−0.13 (−4.93, 4.67)	0.96
Modified BBTD plus losartan versus losartan	1	−7.38 (−9.95, − 4.81)	<0.00001
Modified BBTD plus antihypertensive drugs versus antihypertensive drugs	1	−4.31 (−8.39, − 0.23)	0.04

*Meta-analysis *	3	−4.33 (−8.44, − 0.22)	0.04

**Table 5:** Analyses of diastolic blood pressure.

Trials		MD (95% CI)	*P* value
BBTD versus antihypertensive drugs			
Modified BBTD versus captopril	1	−0.75 (−2.89, 1.39)	0.49

*Meta-analysis *	1	−0.75 (−2.89, 1.39)	0.49

BBTD plus antihypertensive drugs versus antihypertensive drugs			
Modified BBTD plus L-amlodipine versus L-amlodipine	1	1.55 (−2.39, 5.49)	0.44
Modified BBTD plus losartan versus losartan	1	−3.85 (−5.70, − 2.00)	<0.0001
Modified BBTD plus antihypertensive drugs versus antihypertensive drugs	1	−1.24 (−4.04, 1.56)	0.39

*Meta-analysis *	3	−1.57 (−4.54, 1.40)	0.30
